# Long-Term Effectiveness of Scleral Lens Treatment in the Management of Keratoconus: A Systematic Review

**DOI:** 10.7759/cureus.77102

**Published:** 2025-01-07

**Authors:** Adeel Mushtaq, Isaamuddin Alvi

**Affiliations:** 1 Radiology, Liverpool University Hospitals NHS Foundation Trust, Liverpool, GBR; 2 Trauma and Orthopaedics, Oxford University Hospitals NHS Foundation Trust, Oxford, GBR

**Keywords:** corneal ectasia, keratoconus (kc), long-term outcome, scleral contact lens, systematic literature review

## Abstract

This systematic review evaluates the long-term effectiveness of scleral lenses in improving visual outcomes, patient satisfaction, and safety in patients with keratoconus.

A systematic search of six databases (PubMed, Embase, Web of Science, Scopus, Cochrane Library, and MEDLINE) was performed following Preferred Reporting Items for Systematic Reviews and Meta-Analyses (PRISMA) guidelines. The inclusion criteria included studies with more than or equal to three months of follow-up, included a minimum of 20 keratoconus patients, published after year 2020 and outcomes reporting visual acuity, comfort or quality of life. Methodological quality was assessed using the Newcastle-Ottawa Scale and Murad et al. case series tool.

Five studies (463 eyes) met the inclusion criteria. Scleral lenses consistently improved best-corrected visual acuity (BCVA) across studies, with visual gains from baseline logMAR 0.50-0.53 to post-treatment logMAR 0.08-0.09. Significant improvements in vision-related quality of life (National Eye Institute Visual Function Questionnaire (NEI-VFQ) scores) were observed in validated assessments. Complications were infrequently reported but included lens handling issues (10.4%-63%), fogging (58%), and physiological events (e.g., corneal epitheliopathy). Long-term follow-up indicated that 14.6% of the patients experienced worsening visual acuity due to keratoconus progression. Methodological quality was variable, with limitations including variable outcome reporting and limited follow-up duration.

Scleral lenses provide substantial and sustained improvements in visual acuity and quality of life for keratoconus patients, particularly those with advanced disease. However, the evidence is limited by methodological shortcomings and a lack of long-term controlled studies. Future research should prioritise randomised trials with standardised reporting and longer follow-up to better assess complications and durability of outcomes.

## Introduction and background

Keratoconus is the most common primary corneal ectasia. It is a progressive disease characterised by localised corneal thinning, leading to protrusion of the cornea. This commonly occurs in the infero-temporal and central sectors of the cornea; however, superior keratoconus has been described [1]. The reported prevalence of the disease presents a high variability, which can be attributed to the lack of a well-defined diagnostic criteria for keratoconus and heterogeneity of study populations. Population-based studies have reported prevalences ranging from 0.9% to 4.97% [2-11]. Risk factors for the disease include age (particularly within the 20- to 30-year age group), male sex, family history, pre-existing atopic eye disease and connective tissue disease. The role of the environment is theorised to be a complex interplay between genetics, ultraviolet exposure and diet, leading to an increased prevalence within the Middle East and India [5].

The treatment for keratoconus is largely dependent on the severity of the disease. Typically, mild cases are treated with glasses, moderate cases with contact lenses and severe cases may require surgical correction, with the grading as per the Collaborative Longitudinal Evaluation of Keratoconus (CLEK) guidelines [6]. It is estimated that up to 90% of patients with keratoconus are utilising contact lenses for the correction of astigmatism. This can be explained by the fact that the early-phase keratoconus causes regular astigmatism and correction with glasses is used. As the disease progresses in an asymmetrical pattern, glasses can induce anisometropia, increasing visual disturbances in patients and further hampering quality of life [7]. There are several options for corneal irregularity correction, including rigid gas permeable (RGP) contact lenses, piggyback lenses (RGP lens fitted on top of a soft contact lens), soft contact lenses and hybrid lenses (rigid centre with soft hydro-permeable skirt). RGP lenses offer the greatest amount of versatility in managing keratoconus, as the lens design allows for the neutralisation of irregular astigmatism by varying the overlying tear film, thus cancelling out optical aberrance by the disease process. There exist three main designs of RGP lenses: corneal, corneoscleral and scleral. Corneal lenses (CoL) are considered the gold standard RGP lens design and usually are deployed as first-line, with the other designs being utilised in cases of treatment failure. CoLs sit on top of the cornea and thus have the smallest footprint of the three RGP lenses available. Due to this, CoLs exhibit the greatest degree of postural instability, and as a result, require steeper than standard central curvature leading to displacement of the lens weight to the periphery of the cornea and contributing to corneal chronic trauma and worsening keratoconus [8]. There are three main fitting procedures used for CoLs; apical bearing, apical clearing and three-point touch. Apical bearing involves providing a majority of the lens support from the apex of the cornea, where the central zone of the lens ‘bears’ on the cornea. While this provides good visual outcomes, it has been shown to increase corneal scarring [9]. Apical clearing involves providing the primary lens support from the paracentral cornea; however, this is no longer in use due to the poor visual acuity and disease progression. Three-point touch allows the contact lens to bear on several points on the cornea including on the apex and the paracentral cornea, allowing for superior visual outcomes [10].

Corneoscleral lenses, rather than only sitting on the cornea itself, share its bearing with both the peripheral cornea and conjunctiva covering the sclera. The main advantage of this design is improved comfort due to decreased bearing on the highly sensitive cornea itself and improved stability of the lens as it can still move, for example, during blinking and provide optimal optics. However, the location of the bearing here also provides the main side effect of the lens; it has the potential to cause limbal compression and lead to a neovascular response.

Scleral contact lenses, in comparison, are rigid contact lenses that cross the corneal limbus and bear on top of the conjunctiva itself. These tend to be utilised when prior lenses fail and can be very useful in the management of advanced keratoconus where they can delay the need for a corneal transplant to achieve acceptable quality of life [11]. However, short-term studies have indicated that due to the tear film seal created at the corneal surface by the lens, there is an increased risk of corneal hypoxia and resultant oedema [12,13].

Despite the increased use of scleral lenses in the management of keratoconus, with recent use in ocular surface disease, there does not seem to be a definitive consensus on the performance of scleral lenses in the management of keratoconus. If proven to be superior to other RGP lens designs, it can open another avenue to optimise visual outcomes for these patients.

Objective

The objective of this review is to systematically review and analyse the long-term efficacy of scleral lenses in improving visual outcomes, patient satisfaction and minimising complications in patients with keratoconus.

## Review

Methods and materials

This systematic review study was registered with the PROSPERO database with the registration ID CRD4202459275 and did not require any approval from an ethics review board or informed written consent from participants. Completion of reporting for this study was ensured by following the 2020 Preferred Reporting Items for Systematic Reviews and Meta-Analyses (PRISMA) guidelines [14], which set the standard for systematic review and meta-analyses.

Inclusion/Exclusion Criteria

The inclusion criteria to ensure the goals of this study were met were as follows: Studies published in the last 24 years (2000 to 2024); studies following up patients for a minimum of three months post-contact lens insertion (to ensure adequate long-term outcomes are included); inclusion of a minimum of 20 patients diagnosed with keratoconus at any severity in the study cohort; studies offering accessible information of pre- and post-lens insertion outcomes for analysis; study outcomes include one of visual acuity, patient-reported outcome measures or comfort/satisfaction scores.

The exclusion criteria for this study were: studies that focused on the performance of a certain branded scleral lens compared to other scleral lenses on the market, studies that involved patient populations with concurrent severe ocular pathologies other than keratoconus or studies without quantifiable long-term outcomes or insufficient outcome reporting.

Search Strategy and Study Selection

The literature search was performed by both authors using PubMed, Embase, Web of Science, Scopus, Cochrane Library and MEDLINE. Keywords across the databases included (“Scleral Lenses” OR “semi-scleral” OR “miniscleral”) AND (“Keratoconus”) AND (“Visual Acuity” OR “Comfort” OR “Efficacy”). We uploaded our searches to Rayyan (Qatar Computing Research Institute, Ar-Rayya, Qatar). The process of screening was performed stepwise; initially, duplicates were eliminated, then titles and abstracts were screened, and finally full-text review was performed for inclusion. Two reviewers independently screened the articles at each stage and conflicts were resolved by discussion between the reviewers.

Evaluating Methodology Quality and Potential for Bias

The Newcastle-Ottawa Quality Assessment Scale [15] was used to assess the methodology quality of the included prospective studies. This tool was selected for its ease of use compared to the Risk Of Bias In Non-randomised Studies of Interventions (ROBINS-I), which has been shown to translate to better inter-rater reliability [16]. We are expecting that there will be a large quantity of case series that either retrospectively or prospectively evaluate treatment success/failure in the treated population. In these cases, we would use the proposed tool by Murad et al. [17] as other tools require the presence of control groups, which would not be present in these studies. We did not numerically score these studies; a qualitative analysis was done using the Newcastle-Ottawa Quality Assessment Scale.

Outcome Measures

Due to the scope of the review, both visual acuity (VA) and patient-reported outcome measures (PROMS) were considered as final measures. Due to the broad heterogeneity of these items, a meta-analysis was not possible in this review.

Data Extraction and Synthesis

We extracted information from each study on the following aspects: study design, patient population, best-corrected visual acuity (BCVA) before lens care, time of first lens-fitting appointment and final follow-up, patient-reported comfort/satisfaction and complications. A final methodological analysis was performed as per Murad et al. [17].

Although there are many ways of quantifying quality of life, the most validated measure for vision-targeted measure of quality of life is the National Eye Institute Visual Function Questionnaire (NEI VFQ), which has been widely used across a variety of ocular diseases such as keratoconus and also used to assess response to treatment where this has been used, objective comparison can be made between studies.

Results

Search Results

**Figure 1:**
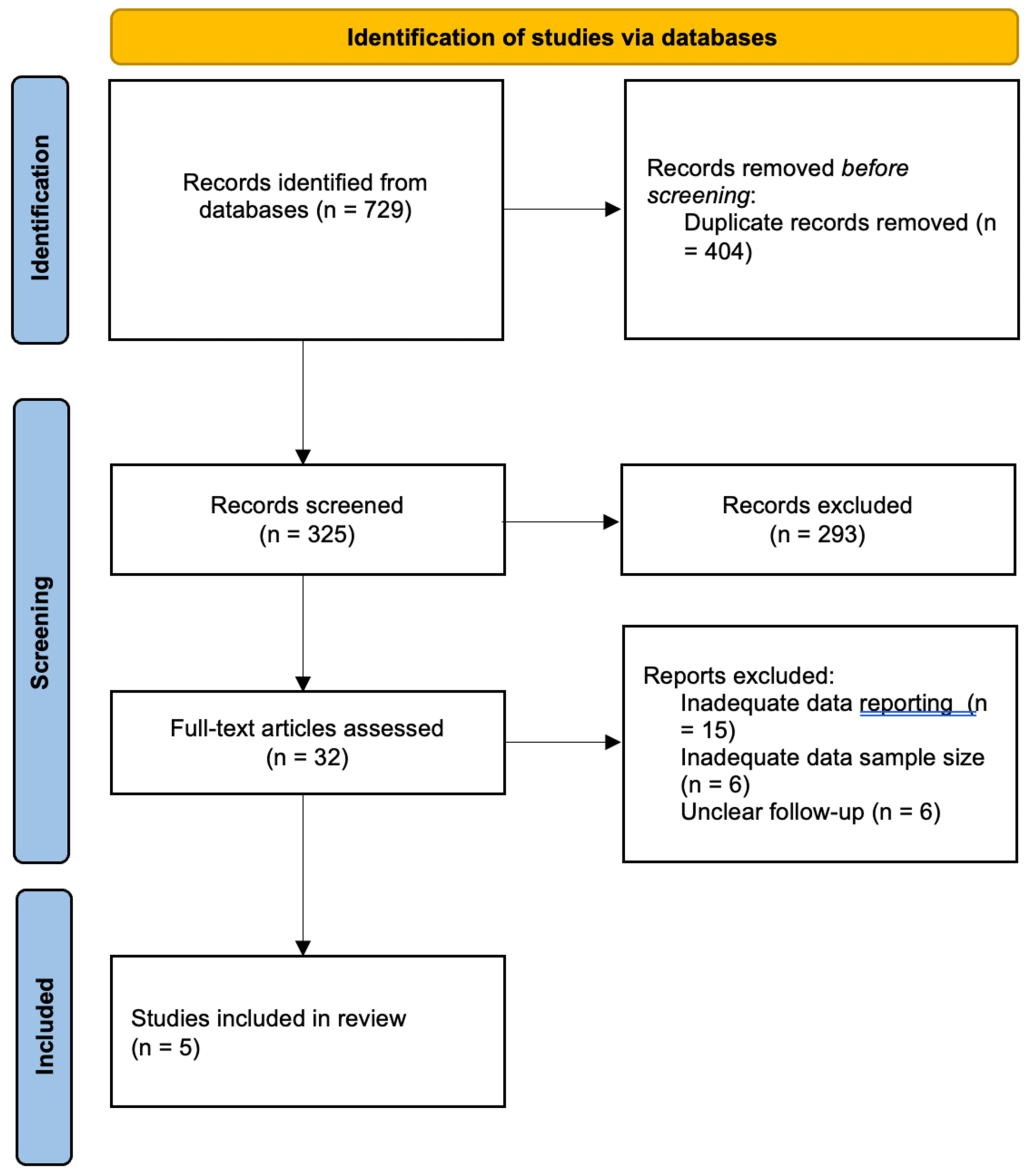
PRISMA flowchart depicting screening process PRISMA: Preferred Reporting Items for Systematic Reviews and Meta-Analyses.

Figure 1 shows the PRISMA flow chart summarising our screening process. Of the initial 729 studies identified, 404 articles were duplicates and it was resolved to leave 325 articles available for title and abstract review. From the initial screening, 293 articles were excluded and 32 were subsequently full text analysed for inclusion. A further 27 articles were excluded in this process, with most of them being excluded due to inadequate data reporting, for example, not separating keratoconus population results reporting from the remaining study population and unclear reporting of one or more study outcomes. Five studies [18-22] involving 463 eyes were finally included for review. Subsequent reference searches from these articles did not yield any studies for inclusion.

Characteristics of Results

Table 1 shows the main characteristics of each study. Three [18-20] out of the five studies were retrospective studies, with Shorter et al. [18] performing a survey-based study upon patients treated for keratoconus with scleral lenses in the public. Three [19-21] studies also measured the keratoconus severity at baseline for the study population using verified scoring systems; however, each used a different scoring system. Overall follow-up duration varied, with Kreps et al. [19] following the cohort up for six months but electing to measure different outcomes (visual acuity and NEI VFQ score) at different times. Those studies that elected to perform a retrospective review had longer follow-up periods, which is most likely due to lower constraints on recruiting participants (no consent required, not time-dependent). As a result, these studies tended to be larger.

**Table 1:** Study design, sample size and duration of follow-up recorded by each included study

Title	Study design	Number of participants	Duration of follow-up
Fuller et al. [21]	Retrospective Study	86 subjects/157 eyes	5 years (2013–2018)
Segal et al. [22]	Retrospective Case Series	30 patients/44 eyes	Mean 17 months (range: 2–96 months)
Shorter et al [18]	Cross-Sectional Survey	76 patients/132 eyes	Years with keratoconus = 21±15
Kreps et al. [19]	Prospective Case Series	50 patients/89 eyes	6 months
Baudin et al. [20]	Prospective Cohort Study	24 patients/41 eyes	3 months

Outcomes and Reporting

Tables 2, 3 outline the visual and patient-specific outcomes reported by the studies respectively. Three [18-20] out of five studies reported initial visual acuity on the logMAR scale, with all these studies initially measuring the best-corrected visual acuity (BCVA) using a Snellen chart and converting it to the logMAR scale for further statistical analyses. Segal et al. [22] measured the visual acuity before and after treatment but only reported BCVA after treatment and only as a frequency of patients who achieved better than 20/40 vision. Kreps et al. [19] reported initial BCVA and BCVA after first fitting, but no indication of there being visual acuity measurements at the end of the study period.

**Table 2:** Visual outcomes reported by each included study Where this data is missing, "-" has been inserted into the entry. ABCD classification was done following Belin MW, Duncan JK. Keratoconus: the ABCD grading system. Klin Monbl Augenheilkd. 2016;233:701–707. 10.1055/s-0042-100626.

Title	Keratoconus stage/severity in population	BCVA before treatment	BCVA after lens fitting	BCVA after final follow-up
Fuller et al. [21]	Mean Severity Score: 3.6±1.0	logMAR 0.50±0.3 (spectacles)	logMAR 0.08±0.1 (unclear if at fitting or follow-up)	Unclear
Segal et al. [22]	-	-	40 eyes (90.9%) achieved VA ≥ 20/40; 52 eyes (94.5%) gained ≥ 2 Snellen lines	Unclear if VA was reassessed at final follow-up
Shorter et al. [18]	-	-	-	-
Kreps et al. [19]	Using ABCD classification: modal A score was 2 (30 eyes, 33.7%); mode B score was 2 (32 eyes, 36%) and modal C score was 1 (33 eyes, 37.1%)	logMAR 0.53±0.21	-	logMAR 0.09±0.10; reported six-month outcome only
Baudin et al. [20]	Krumeich stage 1=8 eyes, stage 2=16 eyes, stage 3=10 eyes and stage 4=7 eyes. Average stage 2.0	-	-	-

**Table 3:** Patient-related outcomes and complications reported from included studies. Where this data is missing, a "-" and been inserted into the entry. NEI-VFQ-25: National Eye Institute Visual Function Questionnaire

Title	Wearing time	Comfort and/or satisfaction	Discontinuation rate and reasoning	Complications reported and frequency
Fuller et al. [21]	-	-	Patients who discontinued were excluded	15 eyes (9.6%) experienced physiological adverse events (e.g., microbial keratitis, hydrops); 87 eyes (55.4%) experienced lens-related adverse events (e.g., deposits, lens breakage); 23 eyes (14.6%) had disease progression worsening visual acuity.
Segal et al. [22]	-	37 patients (86%) reported marked subjective improvement in quality of life	5 patients/7 eyes (10.4%) failure rate due to handling issues or lens discomfort	No significant ocular complications
Shorter et al. [18]	-	Higher satisfaction (3.3/5) and comfort (3.2/5) than gas-permeable (GP) lens wearers	-	56 patients (74%) reported visual difficulties; with 55 (72%) reporting halos, sunbursts and starbursts, and 44 (58%) reporting cloudy or foggy vision. 30 (40%) of ScL wearers had an eye problem requiring a doctor and 36 (47%) reported a problem requiring them to stop wearing their lenses.
Kreps et al. [19]	12 hours/day	Significant NEI-VFQ-39 improvement by average 6.9 points	11 patients (20 eyes) discontinued; 7 patients reported difficulty in lens insertion; 1 patient had visual dissatisfaction and 3 reported discomfort	1 patient had an episode of non-specific corneal epitheliopathy
Baudin et al. [20]	12.3 hours/day	NEI-VFQ-25 improved by 19.5 ± 19.1 points.	-	-

Patient satisfaction and/or comfort when wearing the lenses was reported by all but one of the included studies [21]. Only two of the four studies that investigated this outcome utilised the NEI-VFQ scale, Segal et al. [22] used a questionnaire to measure improvements in quality of life and ocular discomfort and Shorter et al. [18] used a five-point Likert scale to measure their subjective vision, comfort and ease of use of the lenses. Due to similar NEI-VFQ reporting, statistical heterogeneity was calculated to assess whether the results could be pooled for a meta-analysis; however, heterogeneity was very significant, with an I2 of 96%.

All but one study reported adverse events/complications of treatment. Fuller et al. [21] provided a detailed breakdown of all safety events that occurred over the period studied, as this was one of the focuses of the study. Shorter et al. [17] only provided data on lens-related complications as opposed to physiological complications, on which Fuller et al. [21] both distinguished and reported on.

Methodology Analysis

The quality of methodology varied significantly, with most studies demonstrating a clear inclusion of keratoconic populations but lacking rigorous design features to allow for recreation of the study. Prospective studies such as Kreps et al. [19] and Baudin et al. [20] demonstrated clear participant selection, standardised tools for outcome assessment and robust ascertainment from collected data. However, these tended to falter when capturing longer term outcome data as follow-up duration was generally inadequate. This meant that making meaningful long-term conclusions from these studies are difficult.

In contrast, retrospective studies such as Fuller et al. [21] reported larger cohorts (n=86) over a longer duration (five years) but struggled with incomplete reporting issues. In this case, discontinuation rates were ambiguous and worsening visual acuity in 23 eyes (14.6%) due to disease progression was noted without robust causal exploration.

Shorter et al. [18] utilised a survey-based approach, which increased both patients and average time of treatment to 21±15 years. However, the use of five-point Likert scale, when the presence of validated scales exists, makes any real conclusion difficult to compare to other studies. Also, the patient population asked were those who were already instated onto scleral lenses, so there was no consideration for those who tried it and could not tolerate the lens. So there is a high degree of selection bias. Furthermore, the reliance on self-reporting introduces recall bias into this study.

Risk of Bias Assessment

The risk-of-bias assessment, conducted using the Newcastle-Ottawa Scale [15], is outlined in Table 4 and revealed moderate methodological quality across the included studies, with scores ranging from 2/9 to 6/9. Cohort studies generally performed better in the domains of participant selection and outcome assessment, with scores of up to 6/9. However, short follow-up durations and a lack of comparator groups limited their overall rigour. Case-control studies demonstrated a higher risk of bias, particularly in the absence of control matching, reliance on self-reported outcomes, and limited representativeness of cases.

**Table 4:** Risk-of-bias assessment of the included studies using the Newcastle-Ottawa Scale

Study	Selection	Comparability	Outcome	Total score
Fuller et al. [20]	3	1	2	6/9
Segal et al. [21]	2	0	1	3/9
Shorter et al. [17]	2	0	0	2/9
Kreps et al. [18]	3	0	3	6/9
Baudin et al. [19]	2	0	3	5/9

Discussion

Strengths and Limitations of This Review

This systematic review evaluates long-term outcomes of scleral lenses in the management of keratoconus, synthesising evidence from studies with varying designs, methodologies and reporting standards. However, due to its relatively narrow and focused scope, there was a limited amount of literature available on this topic that could be deemed to assess the long-term performance of scleral lenses. This led to a very high degree of heterogeneity, with a mix of retrospective, prospective and cross-sectional studies. While prospective studies provided more robust data through standardised assessment tools and clear inclusion criteria, retrospective studies often suffered from incomplete reporting and selection bias. Survey-based studies, though valuable for assessing patient satisfaction, relied on self-reported outcomes without clinical verification, introducing response bias.

Secondly, the follow-up durations varied significantly across studies, ranging from three months to five years. Short-term studies provided valuable insights into initial improvements in visual acuity and quality of life, but they failed to capture long-term outcomes such as disease progression, complications, and lens discontinuation rates. In contrast, longer-term studies offered critical data on adverse events and visual acuity maintenance but often lacked granular details on patient satisfaction or quality-of-life metrics. This imbalance in follow-up duration limited the ability to draw comprehensive conclusions about the durability of scleral lens performance.

The sample sizes of the included studies also varied widely, with some studies enrolling fewer than 20 patients, while others reported larger cohorts. Smaller sample sizes reduce the generalisability of findings and limit statistical power, making it difficult to draw firm conclusions. Furthermore, confounding factors such as variations in keratoconus severity, prior treatments and lens-fitting protocols were not consistently addressed or adjusted for, introducing a potential bias.

Despite the aforementioned limitations, the studies included in this review do collectively demonstrate that scleral lenses play a crucial role in the long-term management of keratoconus by improving both visual acuity and the quality of life of the patient.

Across studies, scleral lenses were shown to provide substantial improvements in best-corrected visual acuity (BCVA). For example, Fuller et al. [21] reported a significant reduction in logMAR BCVA from 0.50 (spectacles) to 0.08 with scleral lenses, which was sustained over a five-year period. Similarly, Kreps et al. [19] demonstrated significant visual gains, with logMAR BCVA improving from 0.53±0.21 to 0.09±0.10. These findings highlight the ability of scleral lenses to optimise visual performance in keratoconus patients, particularly those with advanced corneal irregularity who are intolerant to other lens modalities.

These findings have been echoed by other published reviews in scleral lens therapy. For instance, a review by Schornack [23] emphasised that scleral lenses offer superior visual rehabilitation compared to corneal gas-permeable lenses in advanced keratoconus, largely due to the lens' ability to vault the irregular corneal surface. Another review by Santodomingo-Rubido et al. [24] corroborated these findings, highlighting that scleral lenses provide stable and improved BCVA in severe keratoconus, with fewer fluctuations in vision compared to soft or hybrid lenses.

However, the long-term maintenance of these visual improvements remains uncertain due to inconsistent reporting in follow-up assessments. Notably, Fuller et al. [21] reported that 23 eyes (14.6%) of participants experienced worsening visual acuity due to disease progression, underscoring the need for vigilant monitoring of keratoconus severity over time.

Quality-of-Life Improvements

Improvements in vision-related quality of life (VRQoL) were consistently reported, particularly in studies using validated tools like the NEI-VFQ-39 and NEI-VFQ-25 questionnaires. Kreps et al. [19] reported that NEI-VFQ scores showed marked improvements across multiple domains, including visual functioning and socio-emotional well-being (e.g., Visual Functioning Scale improved from 21.67 to 23.52; Role Difficulties from -2.10 to -4.04). Similarly, Baudin et al. [20] found that NEI-VFQ-25 scores improved by an average of 19.5 points, reflecting significant enhancements in daily functioning and emotional health.

This is something that is reinforced in other reviews covering this topic. Rathi et al. [25] demonstrated that scleral lenses significantly improve patient-reported outcomes, including visual independence, confidence and emotional well-being, particularly in patients who have failed other treatment modalities. These findings emphasise the role of scleral lenses in not only restoring vision but also enhancing functional and psychosocial quality of life.

However, the short follow-up periods in these studies limit the understanding of whether such improvements persist over the long term. Longer-term studies are required to assess the sustainability of these outcomes, particularly in patients with progressive keratoconus.

Complications and Discontinuation Rates

While scleral lenses generally demonstrate favourable safety profiles, complications and discontinuation rates remain underreported in the literature. Lens-handling issues and discomfort were commonly noted, particularly in studies like Segal et al. [22], where five patients (10.4%) of participants discontinued lens wear due to handling difficulties or discomfort. Shorter et al. [18] highlighted that a large proportion of patients who required scleral lens therapy experienced some sort of visual problem (56 patients; 74%) with a majority (55 patients; 72%) reporting haloes, sunburst and starburst patterns within their visual axis. Furthermore, very importantly, 30 (40%) patients required a doctor due to a lens-related complication and 36 patients (47%) reported having to discontinue wearing their lenses temporarily due to a complication. 

Limitations Due to the Lack of Literature

A major limitation encountered in this systematic review is the lack of high-quality, long-term studies investigating scleral lens outcomes in keratoconus. The absence of randomised controlled trials (RCTs) and comparator studies limits the strength of the evidence available. Most of the included studies were observational, with significant heterogeneity in methodology, follow-up duration and outcome reporting.

Additionally, while improvements in visual acuity and quality of life are well-documented in the short term, few studies provide data beyond 12 months. This gap precludes a comprehensive understanding of the durability of these outcomes, the progression of keratoconus and the long-term safety of scleral lens wear. The underreporting of complications and discontinuation rates further limits the ability to assess the real-world performance and tolerability of scleral lenses.

## Conclusions

This systematic review demonstrates that scleral lenses are a valuable intervention for the management of keratoconus, providing substantial improvements in visual acuity and vision-related quality of life, particularly in patients with advanced disease or intolerance to other lens modalities. However, this review has also highlighted the lack of good quality evidence that has been produced that can help guide clinicians and patients in choosing their preferred treatment method for keratoconus. To achieve this, randomised control trials focusing on the comparison of the different lens types among each other can allow for greater diversity of evidence. Furthermore, a standardisation of reporting would allow for multiple study findings to be compared.
